# Predicting individual emotion from perception-based non-contact sensor big data

**DOI:** 10.1038/s41598-021-81958-2

**Published:** 2021-01-27

**Authors:** Nobuyoshi Komuro, Tomoki Hashiguchi, Keita Hirai, Makoto Ichikawa

**Affiliations:** 1 Institute of Management and Information Technologies, Chiba University, 1-33 Yayoi-cho, Inage-ku, Chiba, 263-8522 Japan; 2 Faculty of Engineering, Chiba University, 1-33 Yayoi-cho, Inage-ku, Chiba, 263-8522 Japan; 3 Department of Imaging Sciences, Faculty of Creative Engineering, Graduate School of Science and Engineering, Chiba University, 1-33 Yayoi-cho, Inage-ku, Chiba, 263-8522 Japan; 4 Department of Psychology, Devision of Behavioral Science, Faculty of Letters, Chiba University, 1-33 Yayoi-cho, Inage-ku, Chiba, 263-8522 Japan

**Keywords:** Engineering, Mechanical engineering

## Abstract

This study proposes a system for estimating individual emotions based on collected indoor environment data for human participants. At the first step, we develop wireless sensor nodes, which collect indoor environment data regarding human perception, for monitoring working environments. The developed system collects indoor environment data obtained from the developed sensor nodes and the emotions data obtained from pulse and skin temperatures as big data. Then, the proposed system estimates individual emotions from collected indoor environment data. This study also investigates whether sensory data are effective for estimating individual emotions. Indoor environmental data obtained by developed sensors and emotions data obtained from vital data were logged over a period of 60 days. Emotions were estimated from indoor environmental data by machine learning method. The experimental results show that the proposed system achieves about 80% or more estimation correspondence by using multiple types of sensors, thereby demonstrating the effectiveness of the proposed system. Our obtained result that emotions can be determined with high accuracy from environmental data is a useful finding for future research approaches.

Emotion estimation has become popular in terms of reforming work style, supporting class, and supporting drivers^1–17^. Lots of emotion estimation researches have been proposed by using multi-modal sensor data^2^. One of the effective approaches to estimate emotion is based on image and video data. Hossain et al. captured speech and image signals of a participant in a smart home scenario for emotion detection^3^. Okada et al. estimated emotion from physiological signals such as RR intervals and blood volumes obtained by analyzing hemoglobin concentrations from facial color images^4^. In Ref.^1^, for supporting classes, students’ emotions are analyzed from classroom videos by recognizing each student’s facial expression. Giannakakis et al. developed a framework to detect stress/anxiety emotions from facial video^5^. Torre et al. developed IntraFace that is an available software package to analyze emotion from facial image data^6^. These works in^4–6^ have shown that the use of facial image data was effective for estimating emotions. However, from the perspective of privacy protection, it is desirable to analyze emotion without the use of camera information.

Wearable devices, which acquire human vital data, have been the other traditional alternative to estimate participant’s emotion. Zamkah et al. discussed anti-stress hormones and cortisol metabolites as the primary stress bio-markers that can be measured by wearable devices^7^. Costa et al. developed the Emotional Smart Wristband to achieve a specific emotion such as calm or excitement^8^. Magno et al. presented an ultra-low-power bracelet and implement multi-layer neural networks for detecting emotion^9^. The emotion analysis method^10–13^ estimates four types of emotions (HAPPY/STRESSED/RELAXED/SAD) from the fluctuations of pulse and skin temperatures. These works in^10–13^ have shown that the use of wearable vital sensor was effective for estimating emotions. Also Kaklauskas et al. developed biometrical, affective, emotional and the surrounding environment maps in shopping by using remote biometrics analysis devices, such as vital sensors and web cameras^14–16^. However, in the emotion analysis method^10–13^, it is necessary for persons to always wear the measurement device, which may limit human behaviors.

The Internet of Things (IoT)-inspired data sensing is also expected for detecting emotion and improving our life. The IoT has been acquiring much attention along with the improvement, miniaturization, and price reduction of wireless devices^18–28^. Many electric devices are connected to the Internet by adhering to the idea of the IoT. The IoT enables physical objects and/or space to communicate with each other. It likewise enables us to obtain various types of environmental data, which can be used for big data analysis. The IoT can also be utilized for various types of applications (i.e., smart home, smart building, smart health care, and smart rearing)^25–28^. From the idea of Society 5.0, as proposed by Japanese Government, combining various types of data obtained using the IoT with machine learning and/or big data analysis enables us to solve social issues^29^. Hong et al. developed a system that estimates humans’ actions (invasion/indoor movement) based on array sensor information^30^. In Ref.^30^, humans’ action is estimated using the Support Vector Machine (SVM). Tao et al. developed a system that predicts the amount of wind power generation by using deep learning^31^. It is expected that the idea of Society 5.0 will enable us to estimate emotions without the need of camera sensors or wearable devices. However, it has been difficult to specify data set in estimating emotions.

This study proposes and builds a customized emotion estimation model for individuals based on collected indoor environment data regarding human perception such as temperature, humidity, light intensity. At the first step, we develop wireless sensor nodes to be used in monitoring working environments. The developed system collected indoor environment data regarding human perception via the Wireless Sensor Network (WSN) and emotions through the system of Ref.^10^. The developed system collects indoor environment data and the emotions data as big data. Then, the proposed system estimates individual emotions without image data from camera sensors or vital data from wearable sensors. In addition, this study investigates whether sensory data are effective for estimating individual emotions. Indoor environmental data obtained by developed sensors and emotions data obtained from vital data are logged over a period of 60 days. Emotions are estimated from indoor environmental data by machine learning method. The experimental results show the effectiveness of the proposed system.

## Methods

### Proposed system structure

**Figure 1:**
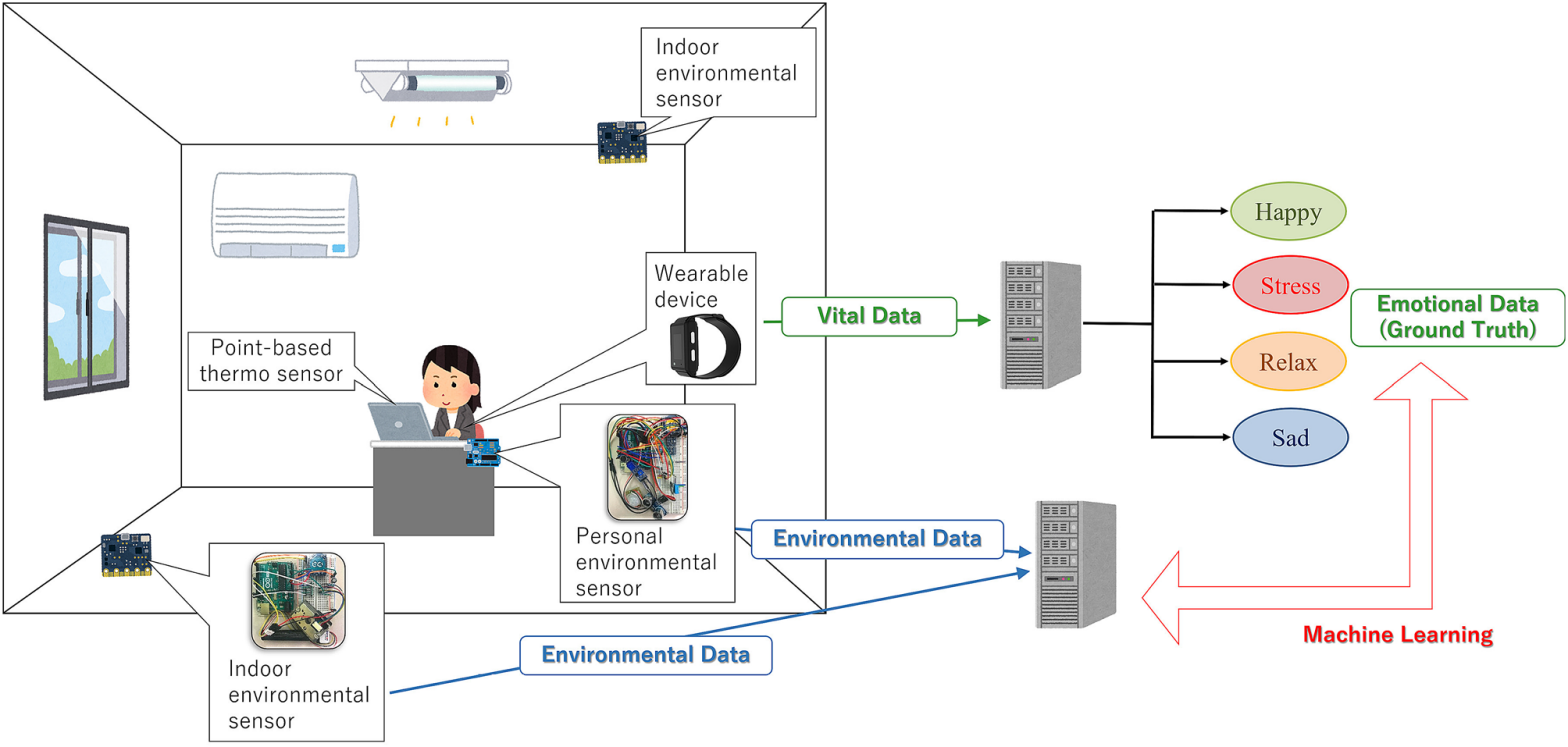
Individual emotion estimation from perception-based sensor data.

Figure 1 shows the structure of the proposed system. The proposed system collects and saves indoor environment data, and sensor nodes measure environmental data regarding human perceptions. Thereafter, sensor nodes send the measured data to the coordinator node. The coordinator node then transfers the received data from the sensor nodes to the data logger. The data logger then logs the data from the sensor node and sends them to the cloud server. Vital and emotion data obtained using the NEC Emotion Analysis Solution^10^ are saved on the cloud server, which are then used as correct answer data for machine learning.

Individual emotions are estimated from obtained indoor environmental data. Emotions are estimated using machine learning method. At the data collection phase, indoor environment data are collected from the developed personal and indoor environment sensors. The phase of estimating emotions from vital sensors draws on the report in^10–12,38^. The emotion analysis system proposed in^10–12,38^ functions by obtaining emotions from the fluctuations of the pulse and skin temperature based on the knowledge of fluctuation analysis of biological signals. The Emotion Analysis System analyzes the balance between the sympathetic nerve and the parasympathetic one the measured skin temperature and heart rate. Then the arousal and valence levels are determined based on the analysis results. It then classifies into following four types of emotions based on the obtained levels: HAPPY, STRESSED, SAD, and RELAXED.

In order to investigate which machine learning method is suitable for the proposed system, we pretested the estimation correspondences of three machine learning methods: SVM, K-Nearest Neighbor (KNN), and random forest. Tables 1, 2, and 3 show the setting parameters for SVM, KNN, and random forest, respectively. These parameters were obtained by grid searching. Table 4 shows the correspondences of SVM, KNN, and random forest, which were obtained by a leave-one-out cross-validation test. In Table 4, nine types of sensors were used. The average correspondence is 74.7% for SVM, 82.1% for KNN, and 86.7% for random forest by using multiple sensor types. Since the random forest algorithm can achieve the highest correspondence, the proposed system estimates emotions with the random forest algorithm. To create the decision tree of the random forest algorithm, the proposed system makes use not only of collected environmental data from personal and indoor environment sensors but also emotions data from NEC Emotion Analysis Solution, which is used as the correct answer. Environment sensor data are linked with individual emotions obtained from emotion analysis method^10–12^, which are measured during working in the experiment room. At the development phase, first, random samples are selected from the collected data set. Next, a decision tree is created and grown for every sample. Estimation results are obtained from every decision tree. At the emotions estimation phase, measured sensor data are encoded. Thereafter, prevailing data on emotions is selected through a majority decision. A decision tree is created for each person. The emotion of each person is then estimated from the decision tree.

**Table 1:** Setting parameters for SVM.

Norm used in the penalization, penalty	l2
Loss function	Squared_hinge
Regularization parameter, *C*	1
Dual	True
Tolerance for stopping criterion, tol	$10^{-4}$
Randomness of the estimator, random_state	None
Kernel type	rbf
Kernel coefficient for rbf, $\gamma$	$\frac{1}{n\_features \times X.var()}$

**Table 2:** Setting parameters for KNN.

Number of neighbors, n_neighbors	6
Weight function used in prediction, weights	Uniform
Algorithm used to compute the nearest neighbors, algorithm	Auto
Leaf size passed to BallTree or KDTree, leaf_size	30
The distance metric to use for the tree, metric	Minkowski
Power parameter for the Minkowski metric, *p*	2

**Table 3:** Decision tree parameters of the proposed system.

Criteria for measuring the quality of a split, criterion	Gini
Maximum depth of decision tree	30
Minimum number of samples required to be at a leaf node	1
Minimum number of samples required to split an internal node	2
Number of trees in the forest, n_estimators	30
Randomness of the estimator, random_state	42

**Table 4:** Emotion estimation accuracy for SVM, KNN, and random forest methods.

Person ID Methods	1	2	3	4	5	6	7	8	9	10	Average
SVM	0.711	0.620	0.747	0.700	0.665	0.901	0.844	0.777	0.718	0.787	0.747
KNN	0.857	0.740	0.808	0.800	0.753	0.912	0.883	0.842	0.770	0.840	0.821
Random forest	0.875	0.812	0.852	0.848	0.834	0.925	0.913	0.885	0.854	0.867	0.867

### Sensor nodes

Each sensor node is composed of environmental data measuring sensors, a wireless sensor module (XBee), and a one-board microcomputer (Aruidno). The operation of sensor nodes was carried out using the one-board microcomputer. Each sensor acquires outage voltage according to the measured value. The one-board microcomputer converts the obtained voltages to corresponding environmental data values, which are temperature (Degree Celsius), humidity (%), illuminance (LUX), loudness (dB), light intensity (LUX), quantized odor level (1 to 1023), distance (cm), CO2 concentration (PPM), dust concentration (μgm^3^), and atmospheric pressure (hPa).

**Figure 2:**
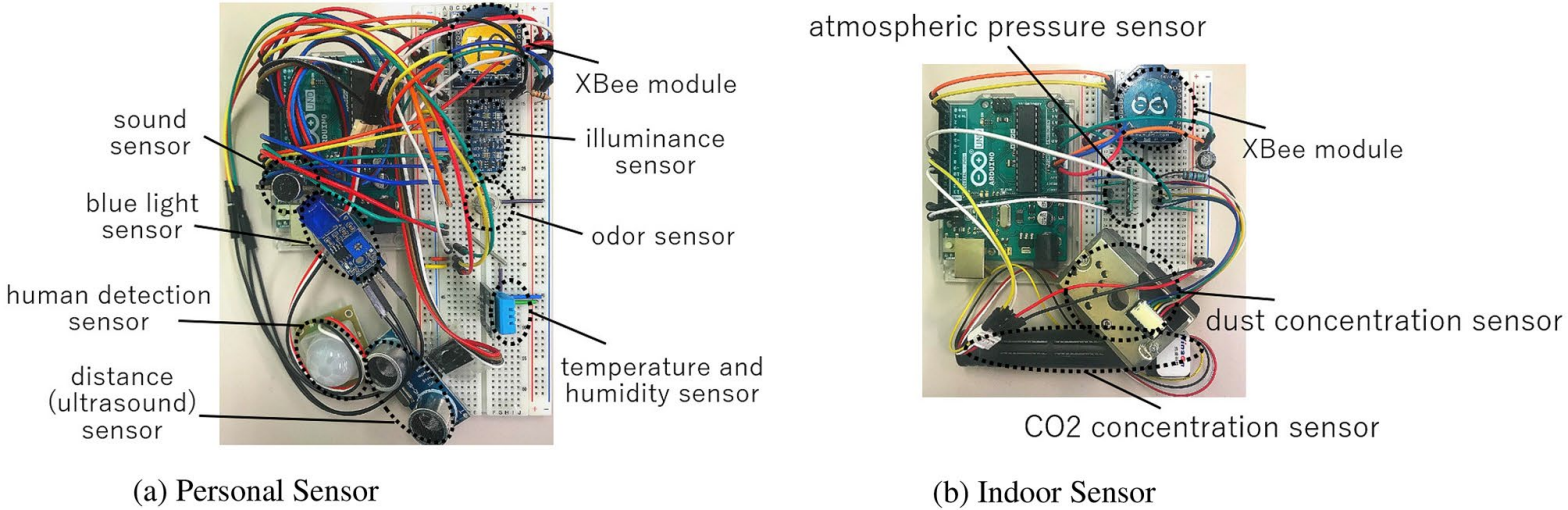
Developed sensors.

Each sensor node measures the indoor environment data periodically. In this study, we developed personal, indoor environment, and thermography sensors in order to measure environment data regarding human perception. The developed personal and indoor sensors are shown in Fig. 2. Personal sensors include temperature and humidity sensors (DHT11), illuminance sensors (TSL2561), blue light intensity sensors (LM393), sound sensors (DFR0034), odor intensity sensors (TGS2450), distance sensors (HC-SR04), and human detection sensors (SE-10). Indoor environment sensors include CO2 concentration sensors (MH-Z16), dust concentration sensors (GP2Y1010AU0F), and atmospheric pressure sensor (BME280). Finally, point based thermo sensors pertain to the infrared array sensor (AMG8833). A point based thermo sensor measures temperature around the sensor and sends the measured data (Degree Celsius) as 8x8 points data. Point based thermo sensors are used for measuring humans’ surface temperature. The server saves the collected data as CSV files. The files include measured data, sensor ID, and sensor data reception time. The details on the construction of the proposed system are described in Ref.^32^.

### Data measurement using sensor nodes

Environmental measurement devices were composed of the developed sensors, a one-board microcomputer, and the XBee router. The star topology sensor network was constructed in two experimental rooms. There are three coordinator nodes, seven personal sensor nodes, two indoor environment sensor nodes, and two point based thermo sensor nodes in each room. Ten personal sensor nodes were placed around ten persons. Point based thermo sensor nodes were placed in front of Person 1 and Person 4. This study was performed in accordance with relevant guidelines and regulations. All participants gave written informed consent, and this study was approved by Chiba University.

Table 5 shows the information of the equipped sensors of each node. Each sensor node measures the environment every 10 s. The experiment was conducted over a period of 60 days.

**Table 5:** Equipped sensors of each node.

ID	Equipped sensor
1–14	Temperature, Humidity, Illuminance, Blue Light Intensity, Sound
	Odor Intensity, Distance, and Human Detection
21–24	Atmospheric Pressure, CO2 Concentration, and Dust Concentration
31–34	Point based Thermo Sensor

### Emotion estimation

The proposed system estimates emotions from environmental data. In particular, the proposed system does not use image data and vital data. Environmental data (i.e., temperature, humidity, illuminance, blue light intensity, loudness, odor intensity, human detection, distance, CO2 concentration, dust concentration, point based thermo sensor, and atmospheric pressure) were logged over a period of 60 days. From the logged environmental data, 70% were used as training data, and the remaining 30% were used as test data.

## Results

We conducted the results by Python language with the scikit-learn library. We obtained the results within several seconds by Intel Core i5 CPU.

### Estimation correspondence of emotions

Table 6 shows the ratio of each emotion estimation correspondence of ten persons, which was obtained by a hold-out test. The ratio of each emotion is defined as the ratio of the number of times each of them appeared to the total number obtained in the experiment.The emotions were estimated from nine types of sensors. Table 6 indicates that emotions estimation correspondence of 8 out of 10 subjects achieved over 80% and that of the remaining two ones achieved over 75%.

**Table 6:** Ratio of each emotion and estimation correspondence of a person.

Person ID	1	2	3	4	5	6	7	8	9	10
Number of data	14420	15244	13226	6685	5849	8154	3606	5801	1843	4781
Happy ratio	0.666	0.396	0.242	0.679	0.347	0.102	0.798	0.779	0.729	0.200
Stress ratio	0.221	0.528	0.723	0.240	0.623	0.876	0.160	0.188	0.236	0.771
Relaxed ratio	0.095	0.044	0.015	0.071	0.021	0.003	0.034	0.029	0.034	0.008
Sad ratio	0.018	0.032	0.020	0.011	0.009	0.019	0.008	0.004	0.002	0.021
Train correspondence	0.999	0.999	0.999	0.998	0.999	0.999	0.998	0.999	0.998	0.999
Estimation correspondence	0.822	0.803	0.832	0.821	0.770	0.905	0.857	0.846	0.775	0.841

In order to confirm the absence of difference between the measured data and estimated data for relatively small data size, we calculated the Bayes factors^39^ under the hypothesis that these data are different (BF${}_{10}$), and obtained 0.328 by the use of *Bayesian t test* in terms of JASP^40^. This value of the Bayes factor is within the level of the moderate evidence for H${}_0$ (below $\frac{1}{3}$)^41^; the measured data is not different from the estimated data.

Table 7 shows the confusion matrices, which were obtained by a hold-out test. Table 7 shows that the appearance ratio of Happy or Stressed is relatively high, while that of Relaxed or Sad is relatively low. Table 7 also shows that the behavior of each emotion ratio differs from person to person.

**Table 7:** Confusion matrix.

		Estimated value						Estimated value			
		Happy	Stress	Relaxed	Sad			Happy	Stress	Relaxed	Sad
(a) Person 1						(b) Person 2					
Measured value	Happy	2697	107	41	4	Measured value	Happy	673	13	1	0
	Stress	203	1165	8	10		Stress	39	120	0	0
	Relaxed	143	14	106	3		Relaxed	18	1	5	0
	Sad	16	28	2	28		Sad	2	4	1	2
(c) Person 3						(d) Person 4					
Measured value	Happy	534	353	4	1	Measured value	Happy	2752	164	41	2
	Stress	113	2549	1	6		Stress	286	1068	5	3
	Relaxed	35	16	6	3		Relaxed	205	6	93	1
	Sad	5	48	1	12		Sad	21	21	3	13
(e) Person 5						(f) Person 6					
Measured value	Happy	415	199	1	0	Measured value	Happy	71	0	0	0
	Stress	210	811	0	0		Stress	36	1870	0	2
	Relaxed	21	7	4	0		Relaxed	3	1	0	0
	Sad	1	15	0	0		Sad	4	35	0	2
(g) Person 7						(h) Person 8					
Measured value	Happy	673	13	1	0	Measured value	Happy	1271	52	12	0
	Stress	39	120	0	0		Stress	111	231	0	0
	Relaxed	18	1	5	0		Relaxed	45	1	8	0
	Sad	2	4	1	2		Sad	4	2	0	0
(i) Person 9						(j) Person 10					
Measured value	Happy	373	14	2	0	Measured value	Happy	163	99	0	0
	Stress	49	84	0	0		Stress	51	1055	0	2
	Relaxed	26	0	3	0		Relaxed	6	4	3	0
	Sad	1	0	0	0		Sad	3	21	0	0

**Figure 3:**
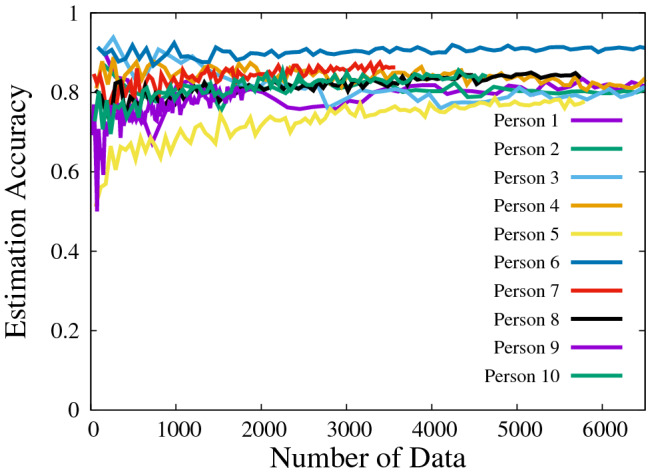
Estimation correspondence versus the number of data.

Figure 3 show the estimation correspondence as a function of the number of data, which was obtained by a hold-out test. The emotions were estimated from nine types of sensors. This figure also indicates that each of estimation correspondence becomes saturated as the number of data increases.

Table 8 shows the estimation correspondence of emotions, which was obtained by a leave-one-out cross-validation test. It also indicates that the estimation correspondence differs according to the types and the number of input sensor data. Using multiple types of sensors improves the estimation correspondence. As presented in Table 8, the estimation correspondence achieved over 80% by using multiple types of sensors. These results show that the WSN-based big data collection is useful for emotions estimation.

**Table 8:** Emotion estimation accuracy versus the number of types of sensors.

Person ID Number of sensors	1	2	3	4	5	6	7	8	9	10	Average
1	0.646	0.533	0.727	0.634	0.629	0.874	0.774	0.780	0.700	0.732	0.703
2	0.685	0.673	0.773	0.754	0.817	0.893	0.871	0.880	0.826	0.814	0.799
3	0.845	0.757	0.829	0.832	0.859	0.904	0.882	0.895	0.862	0.848	0.851
4	0.855	0.800	0.845	0.839	0.842	0.917	0.900	0.889	0.852	0.848	0.861
5	0.869	0.806	0.834	0.850	0.851	0.912	0.903	0.897	0.859	0.866	0.864
6	0.869	0.793	0.835	0.842	0.831	0.912	0.906	0.891	0.856	0.861	0.860
7	0.876	0.806	0.831	0.835	0.824	0.907	0.912	0.891	0.847	0.864	0.859
8	0.871	0.810	0.841	0.842	0.813	0.918	0.916	0.884	0.847	0.863	0.861
9	0.875	0.812	0.852	0.848	0.834	0.925	0.913	0.885	0.854	0.866	0.867
10	0.876	0.806	0.849	0.847	0.835	0.925	0.914	0.884	0.850	0.867	0.865
11	0.872	0.805	0.847	0.847	0.825	0.928	0.909	0.877	0.849	0.864	0.862
12	0.868	0.796	0.844	0.845	0.829	0.927	0.910	0.882	0.855	0.863	0.862
13	0.876	0.794	0.841	0.835	0.826	0.924	0.906	0.876	0.851	0.864	0.860
14	0.873	N/A	N/A	0.819	N/A	N/A	N/A	N/A	N/A	N/A	0.846

Table 9 shows the importance of each sensor type, which was obtained by a hold-out test. The Table 9 indicates that the importance of the CO2 concentration was relatively high for estimating human emotions. The importance of the point based thermo sensor was also relatively high.

**Table 9:** Importance of each sensor.

Person ID	1	2	3	4	5	6	7	8	9	10	Average
CO2 concentration	0.149	0.151	0.158	0.133	0.138	0.162	0.130	0.160	0.180	0.127	0.149
Thermography	0.103	N/A	N/A	0.083	N/A	N/A	N/A	N/A	N/A	N/A	0.093
Temperature	0.019	0.100	0.095	0.099	0.104	0.067	0.058	0.108	0.106	0.078	0.086
Distance	0.101	0.076	0.090	0.079	0.071	0.082	0.083	0.076	0.085	0.095	0.083
Visible ray	0.076	0.065	0.060	0.067	0.067	0.109	0.087	0.074	0.088	0.061	0.079
Loudness	0.063	0.069	0.084	0.060	0.070	0.081	0.082	0.077	0.081	0.075	0.076
Illuminance	0.080	0.061	0.055	0.067	0.065	0.108	0.083	0.075	0.068	0.068	0.076
Odor intensity	0.073	0.086	0.102	0.065	0.105	0.061	0.083	0.086	0.032	0.078	0.075
Humidity	0.014	0.067	0.058	0.059	0.074	0.069	0.101	0.076	0.056	0.055	0.069
Infrared ray	0.069	0.059	0.058	0.062	0.061	0.049	0.094	0.060	0.089	0.065	0.069
Blue light intensity	0.085	0.103	0.082	0.076	0.069	0.064	0.057	0.046	0.070	0.073	0.067
Dust concentration	0.062	0.058	0.062	0.062	0.077	0.052	0.059	0.072	0.064	0.053	0.063
Atmospheric pressure	0.068	0.062	0.050	0.046	0.070	0.059	0.053	0.045	0.052	0.087	0.060
Human Detection	0.039	0.043	0.090	0.042	0.028	0.037	0.030	0.046	0.028	0.053	0.039

## Discussions and conclusions

First, we discuss the impact of the emotion estimation correspondence in Table 6. The estimation correspondence of each person was shown to be about 80% or more. This result shows that the developed personal and indoor environment sensors are effective in estimating emotions. Tables 6 and 7 show the ratio of Happy or Stressed is relatively high, while the ratio of Relaxed or Sad is relatively low. Tables 6 and 7 also show that the behavior of each emotion ratio differs from person to person.

Next, we discuss the impact of the number of sample data. Figure 3 shows that the estimation correspondences become saturated as the number of sample data increases. Although the estimation correspondences are unstable at a small number of sample data, the estimation correspondence of each person becomes stable given a larger number of sample data. As also presented in Table 6 and Fig. 3 that the estimation correspondence achieves over 80% given a large number of sample data although the ratio of each emotion fluctuates at the low number of sample data and also the behavior differs from person to person.

Next, we discuss the number of types of sensors. Table 8 shows that the estimation correspondence differs by the types and the frequency of encoded sensor data. If the proposed system uses only a few sensors, it fails to realize high estimation correspondence. The estimation correspondence directly increases with the number of sensors, particularly when the number of sensors is within the range of one to four. The estimation correspondence is shown to be almost saturated when the number of sensors is larger than five. Further, Table 8 shows that there is a possibility that increasing the number of types of sensors possibly causes the estimation correspondence to decrease owing to over-fitting. Therefore, it is important, in terms of estimating emotions, that the proposed system is able to select the types of sensors to be considered. The experimental results indicate that using nine types of sensors achieved the highest estimation correspondence. The results in Fig. 3 and Table 8 show the effectiveness of big data collection of the proposed system.

Furthermore, the importance of each sensor is presented in Table 9. Clearly, the importance of CO2 concentration ranks the highest among the sensors, regardless of the person analyzed. This result implies that the CO2 concentration can affect emotion. Since a point based thermo sensor can obtain the fluctuation of facial temperature, the importance of a point based thermo sensor was also relatively high. Although the importance of other sensor data depends on the persons analyzed, the emotion estimation correspondence of each person was still over 80%.

Lots of literature have reported the relationships between emotion and physical data such as odor, sound, lighting, and CO2 concentration^33–35^. Bombail introduced that conversely odours can also affect animal/human emotions by inducing a stress response^36^. Ayash et al. reported that student emotion and performance in learning environments were affected by illumination intensity and level^37^. Noguchi et al., investigated and found the relationship between the emotional state, respiratory rate, tidal volume, minute ventilation, and CO2 concentration^38^.

Our personal and indoor sensors can measure multi-modal data, including the above odor, sound, lighting, and CO2 physical data regarding emotion. Our measured data and emotion predictions are implicitly supported by such conventional researches.

In conclusion, this study proposed and built a customized emotion estimation model for individuals based on collected indoor environment data regarding human perception. At the first step, we developed wireless sensor nodes to be used in monitoring working environments and emotion estimations. The developed system collected indoor environment data regarding human perception via the WSN and emotions through the system of Ref.^10^. In addition, the developed system integrated indoor environment data with emotion data. Then, the proposed system estimated individual emotions without image data from camera sensors or vital data from wearable sensors. In addition, this study investigated whether sensory data are effective in estimating individual emotions. The experimental results showed that the proposed system achieved about 80% estimation correspondence by using multiple types of sensors, thereby demonstrating the effectiveness of the proposed system.

Our obtained result that emotions can be determined with high accuracy from environmental data is a useful finding for future research approaches. There is also a possibility that the obtained results contribute to build a less stressful environment. These are the contributions of this study to global innovation. Future works include the increase in the number of research subjects, experiments taking into account seasonality, and creating a general estimation model. Also, we will examine whether it is possible to control emotions by changing the surrounding environment.
